# Interleukin 1 Receptor 1 Knockout and Maternal High Fat Diet Exposure Induces Sex-Specific Effects on Adipose Tissue Adipogenic and Inflammatory Gene Expression in Adult Mouse Offspring

**DOI:** 10.3389/fphys.2020.00601

**Published:** 2020-06-23

**Authors:** Pania E. Bridge-Comer, Jasmine F. Plows, Farha Ramzan, Rachna Patel, Thashma P. Ganapathy, Joanna L. Stanley, Mark H. Vickers, Clare M. Reynolds

**Affiliations:** ^1^Developmental Programming Research Group, The Liggins Institute, The University of Auckland, Auckland, New Zealand; ^2^Saban Research Institute, Children’s Hospital Los Angeles, Los Angeles, CA, United States; ^3^UCD School of Public Health, Physiotherapy and Sports Science, University College Dublin, Dublin, Ireland; ^4^Conway Institute/Institute of Food and Health, University College Dublin, Dublin, Ireland

**Keywords:** developmental programming, adipose tissue, inflammation, IL-1R1, high fat diet, maternal diet

## Abstract

**Background:** The global incidence of obesity continues to rise, increasing the prevalence of metabolic diseases such as insulin resistance, dyslipidemia, and type 2 diabetes mellitus. Low-grade chronic inflammation, associated with the obese state, also contributes to the development of these metabolic comorbidities. Interleukin-1-receptor-1 (IL-1R1), a pro-inflammatory mediator, bridges the metabolic and inflammatory systems. In young male mice, deficiency of IL-1R1 (IL-1R1^−/−^) paired with a high-fat diet (HFD) offered beneficial metabolic effects, however in female mice, the same pairing led to metabolic dysfunction. Therefore, we examined the contribution of maternal HFD in combination with IL1R1^−/−^ to metabolic health in adult offspring.

**Methods:** Female C57BL/6 and IL-1R1^−/−^ mice were randomly assigned to a control diet (10% kcal from fat) or HFD (45% kcal from fat) 10 days prior to mating and throughout gestation and lactation. Male and female offspring were housed in same-sex pairs post-weaning and maintained on control diets until 16 weeks old. At 15 weeks, an oral glucose tolerance test (OGTT) was performed to assess glucose tolerance. Histological analysis was carried out to assess adipocyte size and gene expression of adipogenic and inflammatory markers were examined.

**Results:** IL-1R1^−/−^ contributed to increased body weight in male and female adult offspring, irrespective of maternal diet. IL-1R1^−/−^ and maternal HFD increased adipocyte size in the gonadal fat depot of female, but not male offspring. In female offspring, there was reduced expression of genes involved in adipogenesis and lipid metabolism in response to IL1R1^−/−^ and maternal HFD. While there was an increase in inflammatory gene expression in response to maternal HFD, this appeared to be reversed in IL1R1^−/−^ female offspring. In male offspring, there was no significant impact on adipogenic or lipid metabolism pathways. There was an increase in inflammatory gene expression in IL1R1^−/−^ male offspring from HFD-fed mothers.

**Conclusion:** This study suggests that IL-1R1 plays a complex and important role in the metabolic health of offspring, impacting adipogenesis, lipogenesis, and inflammation in a sex-specific manner.

## Introduction

Obesity is quickly becoming the most common chronic health condition globally and is instrumental in the development of co-morbidities such as dyslipidemia, insulin resistance (IR), and type 2 diabetes (Blüher, 2019). Low-grade chronic inflammation is strongly associated with obesity and is thought to be one of the major contributory factors in progression to overt metabolic disease (Saltiel and Olefsky, 2017). There is now clear evidence that exposure to maternal dietary, emotional, and environmental stressors during gestation can predispose offspring to obesity and metabolic dysfunction in later life (Reynolds et al., 2017; McGowan and Matthews, 2018). However, there is limited knowledge around the impact of metabolic inflammation during pregnancy and its effects on the next generation (Segovia et al., 2017).

There has been a robust effort over recent years to understand the molecular mediators involved in metabolic inflammation. Interleukin (IL)-1β has been continuously implicated as a major driving force in the pathogenesis of metabolic disease (Jager et al., 2007; Koenen et al., 2011). Previous studies have demonstrated that deletion of IL-1R1 signaling prevents the onset of IR in a mouse model of diet-induced obesity (McGillicuddy et al., 2011; Finucane et al., 2015). It is notable that this protection is reserved exclusively to younger male mice with detrimental metabolic effects of IL-1R1 depletion observed in older male mice (García et al., 2006; McGillicuddy et al., 2013).

IL-1 signaling during pregnancy has been associated with deleterious effects including increased activation and expression observed in woman with gestational diabetes mellitus [GDM; Katra et al. (2016)] and pre-eclampsia (Siljee et al., 2013). While IL-1 signaling has a functional role during normal pregnancy, particularly in the induction of labor, increased concentrations as a result of infection or obesity-induced meta-inflammation can alter the hormonal balance which is required for maintenance of normal pregnancy (Allport et al., 2001; Terzidou et al., 2006). Additionally, several groups have identified an association between single nucleotide polymorphisms in genes related to IL-1 signaling and increased risk for pregnancy complications (Li et al., 2014; Nair et al., 2014). However, a recent study by our group has identified that IL-1 signaling may be important for maintaining metabolic health in female mice, with depletion resulting in increased adipocyte size and metabolic dysregulation pre, during, and post pregnancy (Plows et al., 2019). A fine balance exists between the inflammatory milieu and metabolic health and is likely altered in response to factors such as age and sex.

Early life-exposure to inflammatory agents such as bacterial lipotoxin results in programming of immune function (Mandal et al., 2013). However, the effect of maternal “meta-inflammation” resulting from obesity and high fat (HF) consumption has not been comprehensively examined within the developmental programming of obesity and metabolic disease paradigm. This study therefore examined the effect of a maternal HF diet during pregnancy and the potential contribution of IL-1 signaling to metabolic programming in adult offspring with a focus on adipose tissue effects.

## Materials and Methods

### Animal Procedures

All animal procedures were approved by the AgResearch Animal Ethics Committee in accordance with the New Zealand Animal Welfare Act, 1999. IL1R1^−/−^ (B6.129S7-IL1R1tm1lmx/J) and C57BL/6J breeding pairs were imported from the Jackson Laboratory, USA, and housed in a animal containment facility at AgResearch, Ruakura, Waikato, New Zealand (22°C, lights on at 06:00 h, off at 18:00 h, humidity at 40–45%, and woodchip bedding). Third-generation female pups were given free access to standard laboratory chow until 10 weeks of age, at which time they were randomly assigned to receive either purified control diet (CD; Research Diets Inc., New Brunswick, USA; #D12450H, 20% kcal from protein, 70% kcal carbohydrate, and 10% kcal fat) or matched (for protein and micronutrient content) HF diet (HFD, Research Diets Inc.; #D12451, 20% kcal from protein, 35% kcal carbohydrate, and 45% kcal fat). This generated four groups (*n* = 8/group) in a balanced experimental design: (1) C57CD (C57BL/6J fed a CD), (2) C57HF (C57BL/6J fed a HFD), (3) IL1CD (IL1R1^−/−^ fed a CD), and (4) IL1HF (IL1R1^−/−^ fed a HFD). Mice began diets 10 days before mating and throughout pregnancy and lactation. At postnatal day 2 (PD2), litters were weighed and sexed using anogenital distance and were reduced to three males and three females to ensure standardized nutrition until weaning. Weaning occurred at PD21 (*n* = 6–8 litters/group). Offspring were housed in same-sex sibling groups and weighed weekly from week 3 to week 16. An oral glucose tolerance test (OGTT) was carried out 1 week prior to cull.

### Oral Glucose Tolerance Test (OGTT)

Offspring were fasted for 6 h and then weighed. The tip of the tail (<1 mm) was cut and the resulting blood was read by glucometer (Optimum Freestyle Neo, Abbott Laboratories, Alameda, CA, USA). Two grams per kilogram of D-glucose (Sigma-Aldrich, NZ) was orally gavaged, and blood glucose concentrations were measured at 0, 15, 30, 60, 90, and 120 min.

### Tissue Collection

Mice were fasted for 6 h and culled by cervical dislocation. A cardiac puncture was performed immediately following death and blood was collected in an EDTA coated tube and centrifuged at 2,000 rpm for 10 min at 4°C. Resulting plasma was stored at −20°C. Organs were dissected, weighed, and snap-frozen in liquid nitrogen and then stored at −80°C or fixed in neutral-buffered formalin for histological analysis.

### Plasma Analysis

Fasting plasma insulin, leptin, and testosterone concentrations were analyzed using commercial mouse-specific ELISAs (UltraSensitive Mouse Insulin ELISA #90080; Mouse Leptin ELISA #90030; Mouse Testosterone ELISA 80552; Crystal Chem., Chicago, IL, USA), according to manufacturers’ instructions.

### Histological Analysis

Gonadal adipose tissue samples were fixed in 10% NBF and were paraffin embedded and sectioned (10 μm) using a Leica RM 2135 rotary microtome (Leica Instruments, Nussloch, Germany). Hematoxylin and eosin (H&E) staining was performed, and sections were mounted using DPX mountant. Slides were visualized under a light microscope, and images captured with NIS Elements-D software (Nikon 800, Tokyo, Japan). Four representative images were taken from each section by an individual blinded to the study groups. A minimum of 100 adipocytes per animal were analyzed. Any cells that were not fully visible were not included in the analysis. Cells that were 150 μm or under were excluded to ensure that stromal vascular cells were not analyzed. Images were analyzed using ImageJ to determine average adipocyte size and adipocyte size distribution per sample.

### Gene Expression Analysis

RNA was extracted from gonadal adipose tissue using TRI Reagent and stored at −80°C. Single-stranded cDNA was prepared using RT^2^ First Strand Kit (SABioscience; Qiagen, Hilden, Germany). The expression of 84 genes relevant to IR was analyzed using the Mouse Insulin Resistance RT^2^ Profiler PCR Array (SABioscience). mRNA expression was quantified by real-time PCR (RT-PCR) on a LightCycler® 480 SYBR Green I Master (Roche Diagnostics, Auckland, New Zealand). To control between-sample variability, mRNA levels were normalized to the geometric mean of a panel of housekeeping genes for each sample by subtracting the Cycle threshold (Ct) of controls from the Ct for the gene of interest producing a ΔCt value. The ΔCt for each treatment sample was compared to the mean ΔCt for control samples using the relative quantification 2-(ΔΔCt) method to determine fold change (Livak and Schmittgen, 2001).

### Statistical Analysis

Statistical analysis was performed using SPSS 24 (IBM, Armonk, NY, USA). Repeated measure ANOVA was performed for the OGTT and weight trajectory data. All other data were analyzed by two-way factorial ANOVA, with genotype and maternal diet as factors. Outliers were assessed as any value greater than 1.5 box-lengths from the edge of each group’s boxplot, and were subsequently winsorized. The Shapiro–Wilk’s test was then performed to assess normality of the data, and Levene’s test was used to assess homogeneity of variances. In the event that one or more groups were not normally distributed and/or homogenous, the data were suitably transformed. Bonferroni *post hoc* tests were performed for multiple comparisons testing between groups when ANOVA found a significant effect. Differences between groups were considered significant at *p* < 0.05. Data are presented as mean ± SEM and graphed using Prism 6 software (GraphPad Software Inc., La Jolla, USA).

## Results

### IL-1R1^−/−^ Increased Glucose Concentrations and Body Weight Irrespective of Maternal Diet in Female and Male Offspring

**Figure 1:**
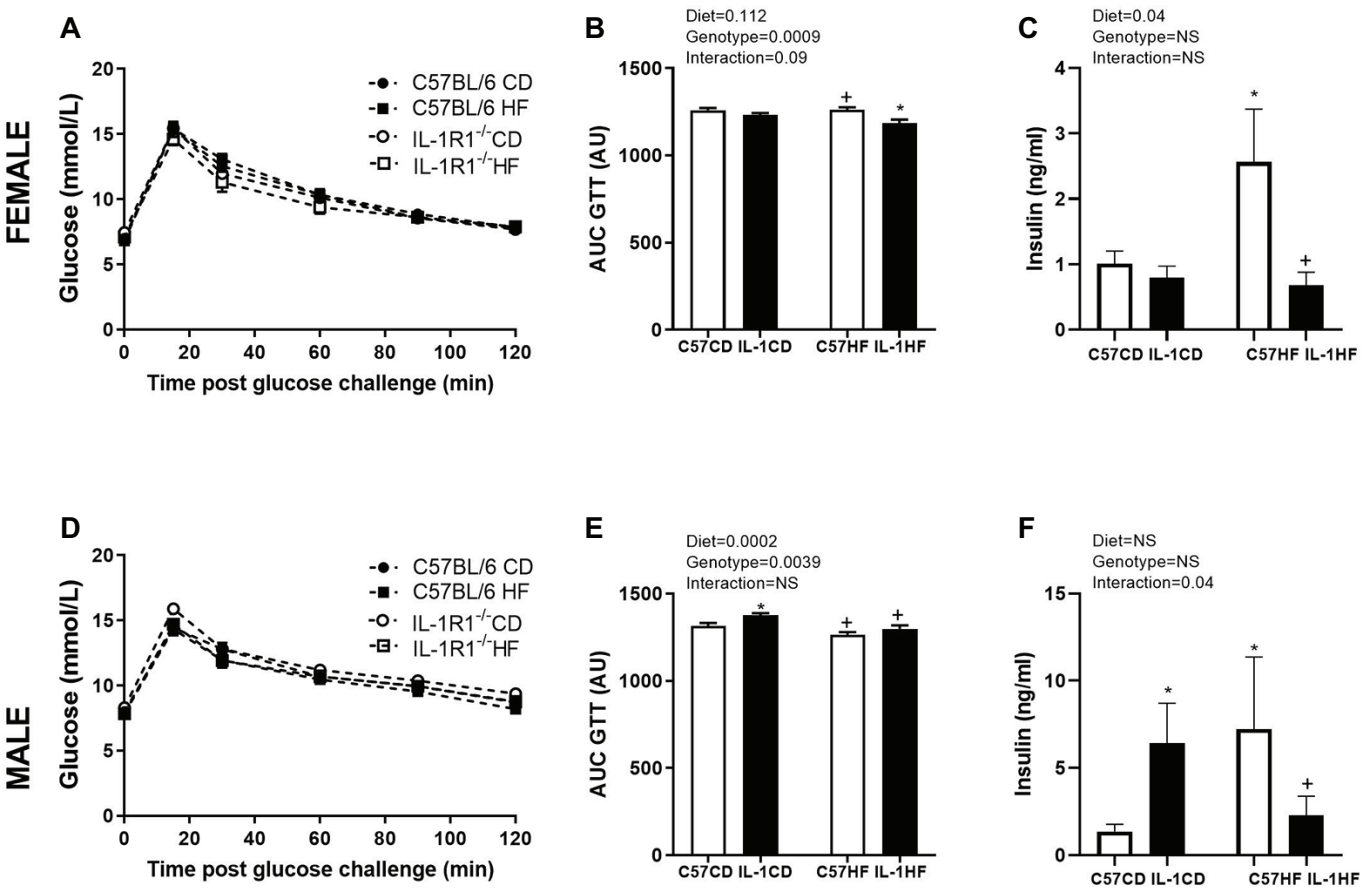
Impact of IL-1R1^−/−^ and maternal high-fat diet (HFD) on offspring glucose tolerance and plasma insulin concentrations. Oral glucose tolerance test (OGTT) (2 g/kg) at 12 weeks in 6 h fasted female **(A)** and male **(D)** offspring. Area under the OGTT curve in female **(B)** and male **(E)** offspring. Insulin concentrations as measured by ELISA in female **(C)** and male offspring **(F)**. Data are expressed as mean ± SEM. *N* = 6–8 litters/group. ^*^p < 0.05 with respect to C57CD. ^+^*p* < 0.05 with respect to C57HF.

IL-1R1^−/−^ induced an increase in the body weight of female and male offspring by 16 weeks of age irrespective of maternal diet. This was associated with a significant increase in fasting plasma glucose and gonadal fat mass (Table 1). There was a reduction in OGTT area under the curve (AUC) in female IL-1HF offspring compared to C57CD and C57HF groups creating an overall genotype effect (Figures 1A,B). In male offspring, there was a significant increase in OGTT AUC in IL-1R1^−/−^ compared to C57BL/6 groups (Figures 1D,E). Maternal diet did not have an effect on weight, fasting glucose, OGTT, or fat mass in male and female offspring. However, there was a maternal diet effect on insulin concentrations in both female and male offspring. In females, C57HF had significantly increased insulin compared to all other groups (Figure 1C). Testosterone concentrations followed a similar pattern with significantly increased concentrations in C57HF compared to all other groups (Table 1). In male offspring, there was a significant increase in insulin concentrations in C57HF compared to C57CD and IL1HF groups and an interaction effect with an increase in IL1CD compared to C57CD and a decrease in IL1HF compared to C57HF (Figure 1F). There was a significant increase in IL1CD testosterone concentrations compared to all other groups (Table 1). There was no significant effect on leptin concentrations between groups (Table 1).

**Table 1:** Physiological outcomes in female and male offspring.

	C57CD	IL1CD	C57HF	IL1HF	Diet	Genotype	DxG
Females
Weight (g)	21.4 ± 0.2	23.4 ± 0.3^*^	22.2 ± 0.4	23.1 ± 0.4^*^	NS	0.0004	NS
Glucose (mmol/L)	7.4 ± 0.4	8.1 ± 0.2	7.4 ± 0.3	8.5 ± 0.4	NS	0.009	NS
Leptin (ng/ml)	1.13 ± 0.3	1.2 ± 0.3	1.11 ± 0.2	0.9 ± 0.3	NS	NS	NS
Testosterone (ng/ml)	0.04 ± 0.01	0.05 ± 0.02	0.18 ± 0.04^*^	0.05 ± 0.01^+^	0.03	0.057	0.043
GF%BW	1.02 ± 0.07	1.46 ± 0.1^*^	1.06 ± 0.07^^^	1.23 ± 0.11	NS	0.017	0.059
Males
Weight (g)	27.8 ± 0.4	31.1 ± 0.7^*^	28.6 ± 0.4^^^	28.6 ± 0.6^^^	0.004	NS	0.003
Glucose (mmol/l)	7.5 ± 0.2	8.3 ± 0.3	7.5 ± 0.3	9.1 ± 0.4^*^^+^	NS	<0.001	NS
Leptin (ng/ml)	1.1 ± 0.3	0.8 ± 0.2	0.8 ± 0.2	0.8 ± 0.1	NS	NS	NS
Testosterone (ng/ml)	0.6 ± 0.2	2.9 ± 0.6^*^	0.7 ± 0.4^^^	1.1 ± 0.4^^^	0.0503	0.005	0.024
GF%BW	1.1 ± 0.1	1.5 ± 0.2	1.3 ± 0.1	1.9 ± 0.2^*^	0.04	0.0006	NS

* *p* < 0.05 with respect to C57CD;

+ *p* < 0.05 with respect to C57HF;

^ *p* < 0.05 with respect to IL1CD.

GF%BW, gonadal fat expressed as a percentage of body weight; DxIL1, interaction between diet and genotype derived from 2-way ANOVA.

### Adipose Tissue Morphology and Adipogenic Gene Expression in Response to Maternal Diet and IL-1R1^−/−^ Genotype

**Figure 2:**
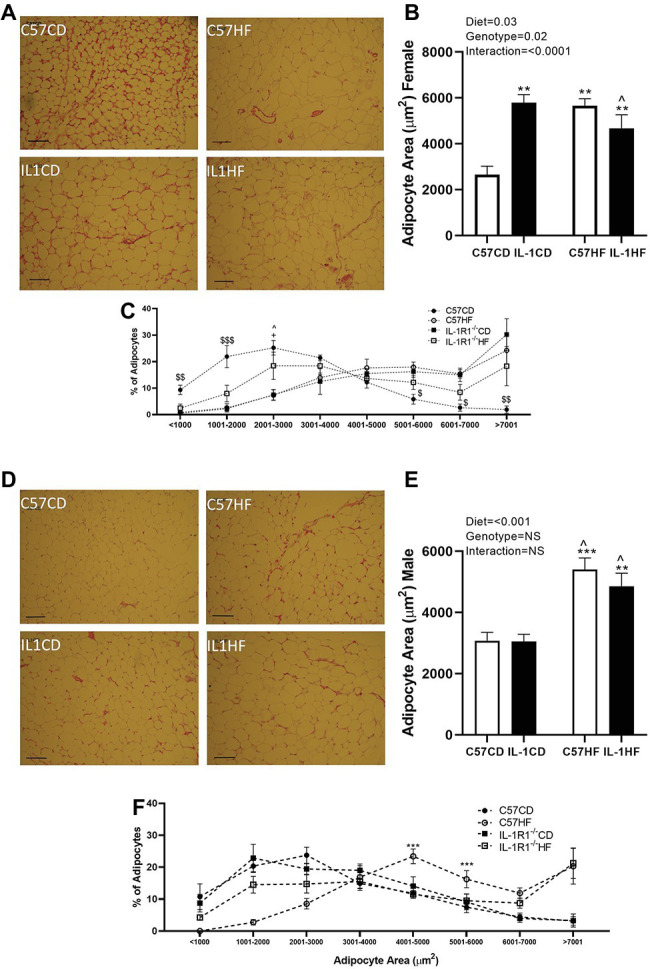
The effect of IL-1R1^−/−^ and maternal HFD on offspring adipocyte size and distribution. Representative gonadal adipose tissue sections stained by hematoxylin and eosin (scale represents 200 μm) in female **(A)** and male **(D)** offspring. Mean adipocyte area analyzed by ImageJ. Four representative images were blindly analyzed per animal in female **(B)** and male **(E)** offspring. Adipocyte area distribution in female **(C)** and male **(F)** offspring. Data are expressed as mean ± SEM. *N* = 6–8 litters/group. ^**^p < 0.01, ^***^p < 0.001 with respect to C57CD. ^+^*p* < 0.05 with respect to C57HF. ^^^*p* < 0.05 with respect to IL1CD. ^$^*p* < 0.05, ^$$^*p* < 0.01, ^$$$^*p* < 0.01, C57CD compared to all other groups.

Maternal diet and IL-1R1^−/−^ resulted in increased average adipocyte size in the gonadal fat pad of female offspring. All groups were significantly increased compared to C57CD. Further, there was a reduction in average adipocyte size between IL1CD and IL1HF groups (Figures 2A,B). When adipocyte size was delineated by size distribution, there was a clear shift to the right in the distribution curve in C57HF, IL1CD, and IL1HF groups compared to C57CD, with a reduction in smaller adipocytes and increased numbers of hypertrophic adipocytes (Figure 2C).

There was a clear effect of maternal diet on adipocyte size in the gonadal fat pad of male offspring although there was no genotype effect (Figures 2D,E). The distribution of adipocyte size showed that C57HF and IL1HF had an increased percentage of adipocytes in the hypertrophic size category (Figure 2F).

**Figure 3:**
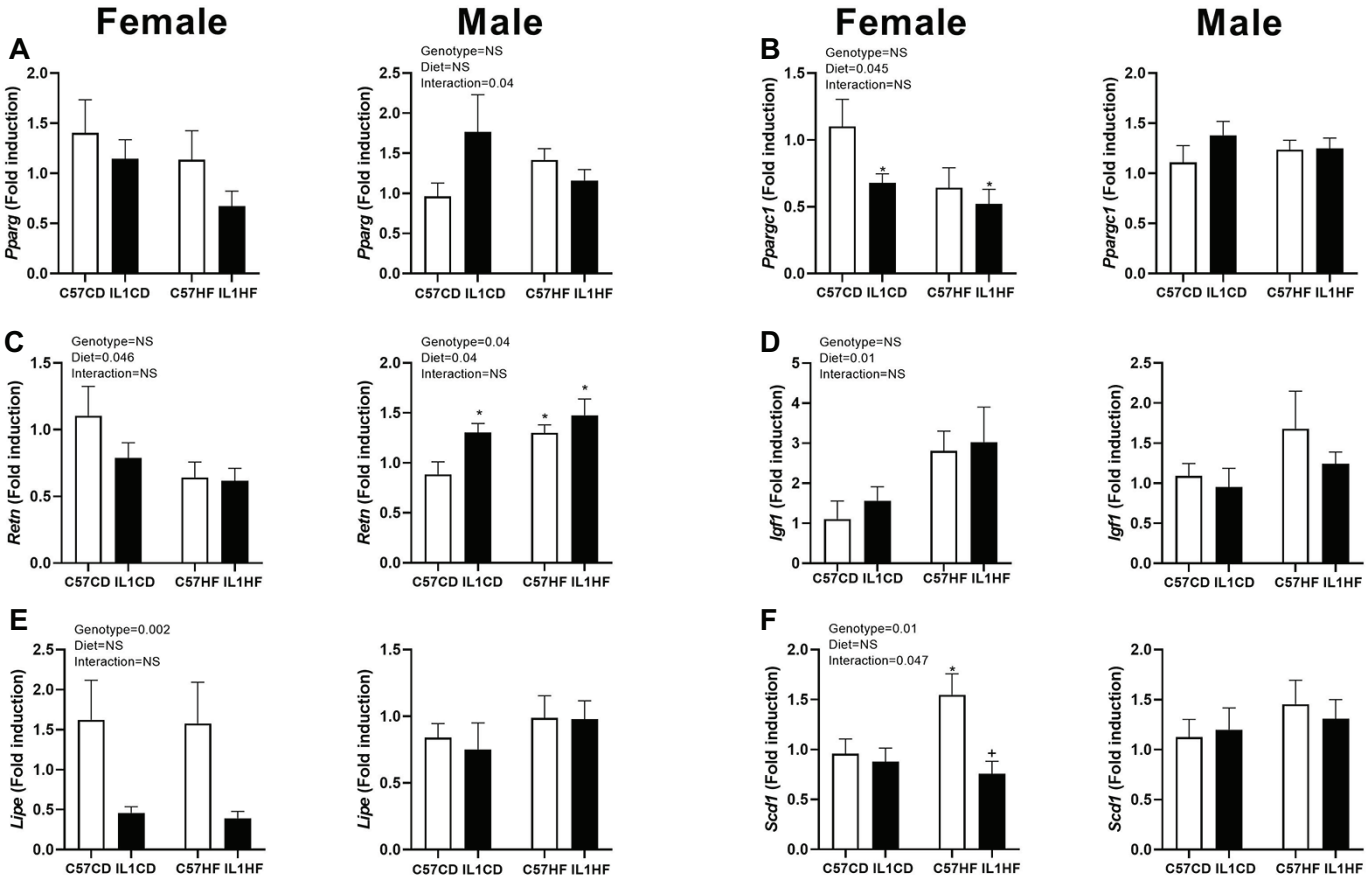
Gene expression of adipogenic markers in adipose tissue. Adipose tissue gene expression of markers related to adipogenesis. **(A)** *Pparg*, **(B)** *Ppargc1*, **(C)** *Retn*, **(D)** *Igf1*, **(E)** *Lipe*, and **(F)** *Scd1*. Data are expressed as mean ± SEM. *N* = 6–8 litters/group. ^*^p < 0.05 with respect to C57CD. ^+^*p* < 0.05 with respect to C57HF.

In line with increased adipocyte size in female offspring, there was a reduction in the gene expression of a number of markers related to adipogenesis. While significance was not reached, there was a trend towards decreased peroxisome profilerator activated receptor gamma (*Pparg*) and resistin (*Retn*) expression in relation to maternal diet and genotype (Figures 3A,C). PPAR co-activator 1 alpha (*Ppargc1*) was significantly reduced with maternal diet with expression reduced in all groups compared to C57CD (Figure 3B). Insulin-like growth factor (*Igf1*) was increased with maternal diet (Figure 3D). There was a significant reduction in hormone sensitive lipase (*Lipe*) in IL-1R1^−/−^ groups irrespective of maternal diet (Figure 3E). Stearoyl Co-A desaturase (*Scd1*) expression was significantly increased in C57HF compared to all other groups (Figure 3F).

In the male offspring, IL1CD had increased *Pparg* expression compared to other groups creating an interaction effect (Figure 3A). *Retn* expression was increased in all groups compared to C57CD (Figure 3C). There was no significant difference between groups with *Ppargc1*, *Igf1*, *Lipe*, or *Scd1* (Figures 3B,D–F).

### IL-1R1^−/−^ Genotype Alters Lipid Metabolism Gene Expression in Gonadal Adipose Tissue of Female but not Male Offspring

**Figure 4:**
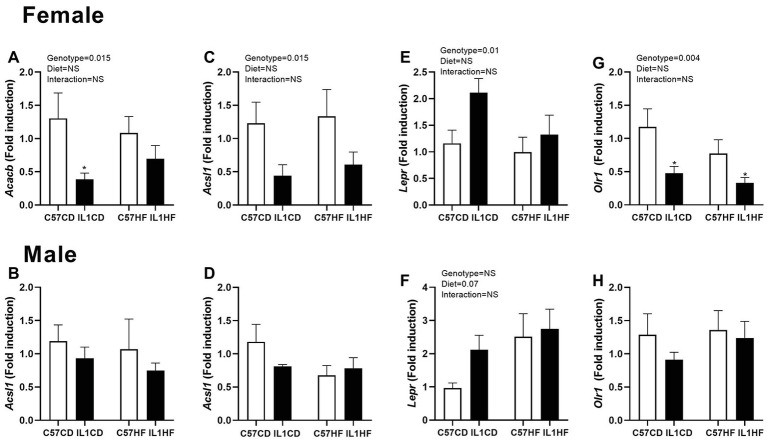
Gene expression of lipid metabolism markers in adipose tissue. Adipose tissue gene expression of markers related to adipogenesis. *Acacb* in female **(A)** and male **(B)** offspring, *Acsl1* in female **(C)** and male **(D)** offspring, *Lepr* in female **(E)** and male **(F)** offspring, and *Olr1* in female **(G)** and male **(H)** offspring. Data are expressed as mean ± SEM. *N* = 6–8 litters/group. ^*^p < 0.05 with respect to C57CD.

In the female offspring, there was a significant reduction in acetyl co-A carboxylase 2 (*Acacb*) gene expression in IL1CD compared to C57CD creating an overall genotype effect (Figure 4A). Long chain fatty acid Co-A ligase 1 (*Acsl1*) expression was reduced in IL-1R1^−/−^ groups irrespective of maternal diet (Figure 4B). There was a significant increase in the leptin receptor (*Lepr*) expression in IL1CD group creating an overall genotype effect (Figure 4E). Oxidized low-density lipoprotein receptor 1 (*Olr1*) was reduced in IL1CD and IL1HF compared to C57CD, creating an overall genotype effect (Figure 4G).

There were no significant effects of either maternal diet or genotype on lipid metabolism markers in the male offspring (Figures 4B,D,F,H).

### Differential Inflammatory Gene Expression Profiles in Female and Male Offspring in Response to Maternal Diet and Genotype

**Figure 5:**
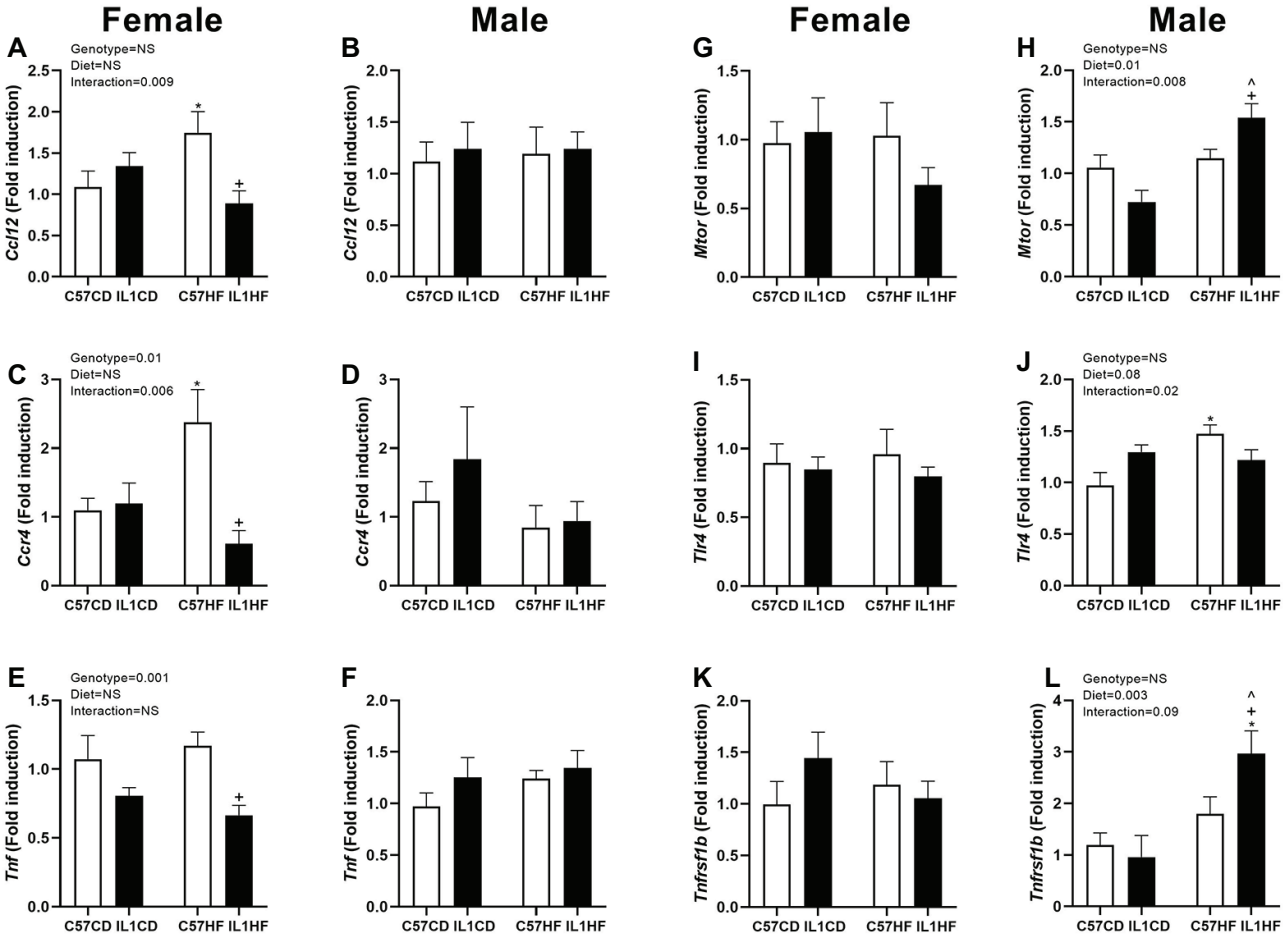
Adipose tissue gene expression of inflammatory markers in female and male offspring. Adipose tissue gene expression of markers related to inflammation. *Ccl12* in female **(A)** and male **(B)** offspring, *Ccr4* in female **(C)** and male **(D)** offspring, *Tnf* in female **(E)** and male **(F)** offspring, *mTor* in female **(G)** and male **(H)** offspring, *Tlr4* in female **(I)** and male **(J)** offspring, and *Tnfrsf1b* in female **(K)** and male **(L)** offspring. Data are expressed as mean ± SEM. *N* = 6–8 litters/group. ^*^p < 0.05 with respect to C57CD. ^+^*p* < 0.05 with respect to C57HF. ^^^*p* < 0.05 with respect to IL1CD.

In the female offspring, there was a significant increase in the monocyte chemotactic protein 5 gene (*Ccl12*) and CC chemokine receptor (*Ccr4*) in C57HF compared to all other groups (Figures 5A,C). There was a reduction in tumor necrosis factor (*Tnf*) expression in IL-1R1^−/−^ groups irrespective of maternal diet (Figure 5E). There was no difference between groups for mammalian target of rapamycin (*mTor*), toll-like receptor 4 (*Tlr4*), and *Tnf* receptor 2 (*Tnfrsf1b*) (Figures 5G,I,K).

In the male offspring, there was no difference between groups for *Ccl12*, *Ccr4*, and *Tnf* (Figures 5B,D,F). *mTor* and *Tnfrsf1b* expressions were significantly increased in the IL1HF group contributing to an overall maternal diet effect. There was a decrease in IL1CD compared to all other groups creating an interaction effect (Figure 5H), however, this only reached significance with *mTor* (Figures 5H,L). *Tlr4* was increased in the C57HF group compared to C57CD. This was partially normalized in the IL1HF group contributing to an interaction effect (Figure 5J).

## Discussion

It is now well-accepted that maternal dietary stressors during pregnancy can have a marked impact on the risk of obesity and cardiometabolic disease in offspring during later life (Reynolds et al., 2017; Chang et al., 2019). Furthermore, the critical windows where early life nutrition and environment influence later metabolic health coincide with the development of the immune system. The IL-1 signaling pathway represents a point of intersection between metabolic and inflammatory systems, with previous work demonstrating a protective metabolic effect of IL-1R1 depletion in young male mice exposed to diet-induced obesity (McGillicuddy et al., 2011). However, it is clear that the role of this pathway in metabolic processes is complex, with evidence that older male mice become spontaneously obese and insulin resistant (García et al., 2006; McGillicuddy et al., 2013). A recent study from our group has also highlighted the detrimental effect of IL-1R1 depletion in nulliparous, pregnant, and postpartum female mice (Plows et al., 2019). Here, we demonstrated a reduction in weight gain and decreased kilocalorie consumption over pregnancy in IL-1R1^−/−^ mice where there was no change in glucose tolerance and an increase in adipocyte size resulting in decreased adipose tissue insulin sensitivity. Given the complexity of this pathway in terms of metabolic regulation, we examined its impact in relation to metabolic programming of offspring adiposity and glucose tolerance. There were minimal changes in glucose tolerance, with protective effects of IL-1R1^−/−^ on maternal HFD-induced programming of insulin concentration in both sexes. Both IL-1R1^−/−^ and maternal HFD had a considerable negative impact on adipocyte size as well as lipid metabolism and adipogenic gene expression in female but not male offspring despite beneficial effects of IL-1R1^−/−^ in inflammatory pathways.

In the current study, female offspring on an IL-1R1^−/−^ genetic background remained heavier and had an increased fat mass. Despite this, maternal HFD-induced increases in insulin secretion were prevented with IL-1R1 depletion, accompanied by a moderate improvement in glucose tolerance. Following a similar trend to insulin, IL-1R1 depletion prevented elevation of maternal-HFD induced testosterone concentrations in female offspring. To our knowledge, there are no data examining the impact of IL-1R1 signaling on testosterone production. However, it is possible that elevated testosterone concentrations in the maternal HFD diet fed control animals may contribute to adverse metabolic outcomes such as hyperinsulinemia. Not only is there evidence implicating testosterone in the pathogenesis of insulin resistance in females (Corbould, 2008) but there are also studies which have demonstrated a developmental origin for polycystic ovary syndrome, characterized by increased testosterone and metabolic dysfunction (Franks, 2012).

There was no effect of maternal HFD on glucose tolerance, weight gain, or testosterone concentrations in male offspring. However, there was a programming effect on insulin concentrations. Despite preventing hyperinsulinemia, male offspring with an IL-1R1^−/−^ background had increased, albeit modestly, glucose intolerance. This was accompanied by a significant increase in weight and fat mass. There is evidence that IL-1R1^−/−^ mice become spontaneously obese and glucose intolerant as they age (García et al., 2006; McGillicuddy et al., 2013). These animals were culled at 16 weeks of age, this may represent a time-point at which this transition to a detrimental metabolic phenotype begins. Given the increase in fat mass in the IL1HF group, it is unlikely that IL-1R1 signaling represents a valid target for the effects of maternal HFD diet on developmental programming of obesity.

In line with overall increases in weight and fat mass, it is unsurprising to see an overall increase in adipocyte size in response to both maternal HFD and IL-1R1^−/−^ background in female offspring. Maternal HFD preempted an increase in adipocyte size irrespective of genotype in male offspring. There is significant evidence showing that adipose tissue dysfunction is related to the enlargement of adipocytes. The ability of adipocytes to differentiate from preadipocytes has a huge impact on the health and function of the adipose tissue. When these adipogenic processes are disrupted, adipocyte hypertrophy occurs. This reduces the capacity of the adipose tissue to respond to insulin contributing to systemic metabolic dysfunction. There are a number of studies which demonstrate that adipocyte hypertrophy in the absence of obesity can predict later type 2 diabetes (Weyer et al., 2000; Hammarstedt et al., 2012). The converse of this is also true where severely obese individuals with smaller adipocytes remain insulin sensitive (Klöting et al., 2010).

A panel of genes relating to adipogenic processes were examined to determine the relationship between adiposity and metabolic health in response to maternal HFD and IL1R1^−/−^. There was a significant reduction of *Ppargc1*, which encodes PGC-1alpha, a master regulator of mitochondrial biogenesis, in response to both IL-1R1^−/−^ genotype and maternal HFD in female but not male offspring. PGC-1alpha acts as a co-activator for PPARγ and is critical for adipogenesis and the regulation of fatty acid oxidation pathways (Liang and Ward, 2006). Reduction of *Ppargc1* in both morbidly obese patients (Semple et al., 2004) and rodents fed a HFD (Kleiner et al., 2012) is associated with significant metabolic dysregulation. It is therefore likely that reduction in this co-activator prevents adipocyte expansion resulting in hypertrophy, thus limiting the capacity to both store and efficiently process fatty acids. In the current study, we also see a negative correlation between *Ppargc1* and *Igf1* in the female offspring. While IGF1 is a known regulator of growth and differentiation, there is evidence that as adipocytes undergo hypertrophy, there is a reduction in IGF1 resulting in recruitment of macrophages to the adipose tissue, and these immune cells then produce IGF1 to compensate for the reduction in adipocytes (Chang et al., 2016).

Hormone sensitive lipase (HSL) is encoded by the gene *Lipe* and is involved in the mobilization of fat stores through the hydrolysis of lipids to fatty acids. There is evidence that knockout of HSL results in increased adipocyte size in rodents (Osuga et al., 2000) and a loss of function mutation in humans was associated with increased risk for metabolic dysfunction (Albert et al., 2014). These studies underscore the importance of HSL in adipocyte function. The current study demonstrates a significant reduction in *Lipe* in female IL1R1^−/−^ mice irrespective of genotype. There is also a decrease in *Acacb*, which encodes the protein ACC2, a rate limiting step in fatty acid uptake and oxidation in the mitochondria, in female IL1R1^−/−^ groups irrespective of maternal diet. Depletion of this protein improves overall metabolic health in mice fed a HFD (Takagi et al., 2018). Furthermore, there was a decrease in expression of *Acsl1*, which encodes a protein which mediates the conversion of long-chain fatty acids to fatty acyl-CoA esters producing important substrates for mitochondrial fatty acid biosynthesis, in IL1R1^−/−^ groups irrespective of genotype. Recent reports have demonstrated that *Acsl1* depletion dampens insulin stimulated glucose uptake and fatty acid efflux in 3t3-L1 adipocytes (Lobo et al., 2009). Overall, these changes in lipid metabolism may inhibit the efficiency of the adipose tissue to mobilize and efficiently utilize lipids which in the short term may reduce the flux of fatty acids to peripheral tissues but may have long term effect *via* the enlargement of adipocytes and overall lipid metabolic health.

Female offspring also exhibited alterations in the expression of several genes involved in immune cell chemotaxis. *Ccl12* encodes MCP-5, which promotes macrophage recruitment to the adipose tissue and promotes IR in response to HFD (Kim et al., 2014). The current study demonstrated an increase in *Ccl12* gene expression in response to maternal HFD, and IL1R1^−/−^ prevented this increase. There is some evidence that *Ccl12* is increased in response to prenatal exposure to HFD. However, these studies focus on behavioral changes in the offspring and there appears to be a paucity of information on *Ccl12* in relation to metabolic health in the offspring. We also see an increase in the expression chemokine receptor *Ccr4* in response to maternal HFD in C57BL/6 but not IL1R1^−/−^ mice. This receptor binds MCP-1, a key protein in the recruitment of macrophages to the adipose tissue, and subsequent IR in response to diet-induced obesity (Kanda et al., 2006). Our results are perhaps not surprising given that MCP-1 is involved in the regulation of the IL-1 pathway (Gavrilin et al., 2000). Furthermore, a role for this protein has been shown in response to maternal HFD, where offspring from mothers fed a HFD had increased MCP-1 which was associated with improved glucose tolerance (Murabayashi et al., 2013). There is also evidence of an IL1R1^−/−^ mediated reduction in the gene expression of *Tnf*, a potent cytokine known to have a negative influence on metabolic health (Hotamisligil, 1999). It is possible that the moderate benefits in glucose tolerance observed in female offspring, despite adipocyte hypertrophy, may be mediated by alterations in immune signaling.

While the impact of maternal diet and IL1R1^−/−^ on lipid metabolism and chemokine expression is limited in males, there is evidence of alterations in several genes which influence metabolic inflammatory processes. mTOR is a critical regulator of cellular metabolism and plays an important role in adipogenesis, lipid metabolism, and inflammatory processes (Mao and Zhang, 2018). Hyper-activation of mTOR signaling has the capacity to increase fat mass and reduce glucose tolerance in mice (Robitaille et al., 2013). It is therefore possible that the increase in adipocyte size in male offspring from HFD-fed mothers may be linked to increased mTOR expression. We also observed a significant increase in *Tnfrsfb*, a receptor for TNF, in the adipose tissue of male offspring exposed to prenatal HFD, independent of genotype. Given the important role of TNF in metabolic dysfunction and adipose tissue biology, it is possible that increases in this receptor may compensate for the lack of IL-1 signaling and thereby influence the glucose intolerance observed in male offspring.

There are several limitations to this current study. Firstly, the knockout model used represents a whole body knockout of the IL1R1 receptor. This may be problematic when interpreting results in specific tissues as the overall phenotype observed may be due to a complex interplay of different organ systems. It would therefore be useful to develop an adipose tissue specific IL1R1^−/−^ to examine the specific contribution of adipose tissue inflammation in the developmental programming paradigm. Another limitation is the lack of littermate controls. While animals came from the same source and IL1R1^−/−^ were maintained on a C57Bl/6 background, there is the possibility that artifactual variation might be introduced between the C57Bl/6 control group and the IL1R1^−/−^ group. This may result from factors such as differences in microbiome composition, and genetic drift etc. and may ultimately skew the results of the study. It is possible that some of the detrimental effects observed in the IL1R1^−/−^ group are due to compensation in other inflammatory mediators such as TNF. We had a limited capacity to conduct comprehensive analysis of circulating plasma proteins. This limited our capacity to uncover any changes in inflammatory profile of these animals. We did examine the inflammatory profile in adipose tissue *via* PCR analysis, a high correlation between RNA and protein concentrations in key inflammatory proteins.

## Conclusion

Previous work from our group demonstrated that IL-1R1^−/−^ resulted in metabolic dysregulation during pregnancy and demonstrated features associated with gestational diabetes including significant adipocyte hypertrophy and evidence of adipose tissue IR (Plows et al., 2019). We sought to expand this work by examining whether IL-1 signaling impacts long-term outcomes in offspring exposed to a HFD *in utero*. Our results show that while there was little impact on glucose tolerance between groups, both IL1R1 knockout and maternal HFD resulted in adipocyte hypertrophy and altered gene expression in pathways relating to adipogenesis, lipid metabolism, and inflammation in a sex-specific manner. This furthers the understanding of diet-induced metabolic inflammation in relation to *in utero* exposures and lends support to the importance of studying both male and female subjects. There are clear beneficial effects of depletion of the IL-1 signaling pathway in females, particularly in relation to inflammation which are not seen in males. This may in part explain why males are more susceptible to metabolic dysregulation following *in utero* exposure to HFD. This work highlights the complexity of IL-1 signaling in terms of metabolic health and the importance of cardiometabolic outcomes in response to *in utero* exposures.

## Data Availability Statement

The raw data supporting the conclusions of this article will be made available by the authors, without undue reservation, to any qualified researcher.

## Ethics Statement

The animal study was reviewed and approved by AgResearch Animal Ethics Committee in accordance with the New Zealand Animal Welfare Act, 1999.

## Author Contributions

PBC collected data, performed analysis, and contributed to the writing of the manuscript. JP and FR performed analysis and edited the manuscript. RP and TG collected data. JS and MV reviewed the manuscript. CR designed the study, collected data, and wrote the manuscript. All authors have read and approved the manuscript.

## Conflict of Interest

The authors declare that the research was conducted in the absence of any commercial or financial relationships that could be construed as a potential conflict of interest.

## References

[ref1] Albert J. S. Yerges-Armstrong L. M. Horenstein R. B. Pollin T. I. Sreenivasan U. T. Chai S.. (2014). Null mutation in hormone-sensitive lipase gene and risk of type 2 diabetes. N. Engl. J. Med. 370, 2307–2315. 10.1056/NEJMoa1315496, PMID: 24848981 PMC4096982

[ref2] Allport V. C. Pieber D. Slater D. M. Newton R. White J. O. Bennett P. R. (2001). Human labour is associated with nuclear factor-κB activity which mediates cyclo-oxygenase-2 expression and is involved with the ‘functional progesterone withdrawal’. Mol. Hum. Reprod. 7, 581–586. 10.1093/molehr/7.6.581, PMID: 11385114

[ref3] Blüher M. (2019). Obesity: global epidemiology and pathogenesis. Nat. Rev. Endocrinol. 15, 288–298. 10.1038/s41574-019-0176-8, PMID: 30814686

[ref4] Chang E. Hafner H. Varghese M. Griffin C. Clemente J. Islam M.. (2019). Programming effects of maternal and gestational obesity on offspring metabolism and metabolic inflammation. Sci. Rep. 9, 1–15. 10.1038/s41598-019-52583-x, PMID: 31690792 PMC6831633

[ref5] Chang H. R. Kim H. J. Xu X. Ferrante A. W. (2016). Macrophage and adipocyte IGF1 maintain adipose tissue homeostasis during metabolic stresses. Obesity 24, 172–183. 10.1002/oby.21354, PMID: 26663512 PMC4793714

[ref6] Corbould A. (2008). Effects of androgens on insulin action in women: is androgen excess a component of female metabolic syndrome? Diabetes Metab. Res. Rev. 24, 520–532. 10.1002/dmrr.872, PMID: 18615851

[ref7] Finucane O. M. Lyons C. L. Murphy A. M. Reynolds C. M. Klinger R. Healy N. P.. (2015). Monounsaturated fatty acid–enriched high-fat diets impede adipose NLRP3 inflammasome–mediated IL-1β secretion and insulin resistance despite obesity. Diabetes 64, 2116–2128. 10.2337/db14-1098, PMID: 25626736

[ref8] Franks S. (2012). Animal models and the developmental origins of polycystic ovary syndrome: increasing evidence for the role of and rogens in programming reproductive and metabolic dysfunction. Endocrinology 153, 2536–2538. 10.1210/en.2012-1366, PMID: 22610962

[ref9] García M. C. Wernstedt I. Berndtsson A. Enge M. Bell M. Hultgren O.. (2006). Mature-onset obesity in interleukin-1 receptor I knockout mice. Diabetes 55, 1205–1213. 10.2337/db05-1304, PMID: 16644674

[ref10] Gavrilin M. A. Deucher M. F. Boeckman F. Kolattukudy P. E. (2000). Monocyte chemotactic protein 1 upregulates IL-1beta expression in human monocytes. Biochem. Biophys. Res. Commun. 277, 37–42. 10.1006/bbrc.2000.3619, PMID: 11027635

[ref11] Hammarstedt A. Graham T. E. Kahn B. B. (2012). Adipose tissue dysregulation and reduced insulin sensitivity in non-obese individuals with enlarged abdominal adipose cells. Diabetol. Metab. Syndr. 4:42. 10.1186/1758-5996-4-42, PMID: 22992414 PMC3523053

[ref12] Hotamisligil G. S. (1999). The role of TNFalpha and TNF receptors in obesity and insulin resistance. J. Intern. Med. 245, 621–625. 10.1046/j.1365-2796.1999.00490.x, PMID: 10395191

[ref13] Jager J. Grémeaux T. Cormont M. Le Marchand-Brustel Y. Tanti J. -F. (2007). Interleukin-1β-induced insulin resistance in adipocytes through down-regulation of insulin receptor substrate-1 expression. Endocrinology 148, 241–251. 10.1210/en.2006-0692, PMID: 17038556 PMC1971114

[ref14] Kanda H. Tateya S. Tamori Y. Kotani K. Hiasa K. Kitazawa R.. (2006). MCP-1 contributes to macrophage infiltration into adipose tissue, insulin resistance, and hepatic steatosis in obesity. J. Clin. Invest. 116, 1494–1505. 10.1172/JCI26498, PMID: 16691291 PMC1459069

[ref15] Katra P. Dereke J. Nilsson C. Hillman M. (2016). Plasma levels of the interleukin-1-receptor antagonist are lower in women with gestational diabetes mellitus and are particularly associated with postpartum development of type 2 diabetes. PLoS One 11:e0155701. 10.1371/journal.pone.0155701, PMID: 27223471 PMC4880279

[ref16] Kim D. Kim J. Yoon J. H. Ghim J. Yea K. Song P.. (2014). CXCL12 secreted from adipose tissue recruits macrophages and induces insulin resistance in mice. Diabetologia 57, 1456–1465. 10.1007/s00125-014-3237-5, PMID: 24744121

[ref17] Kleiner S. Mepani R. J. Laznik D. Ye L. Jurczak M. J. Jornayvaz F. R.. (2012). Development of insulin resistance in mice lacking PGC-1α in adipose tissues. Proc. Natl. Acad. Sci. U. S. A. 109, 9635–9640. 10.1073/pnas.1207287109, PMID: 22645355 PMC3386123

[ref18] Klöting N. Fasshauer M. Dietrich A. Kovacs P. Schön M. R. Kern M.. (2010). Insulin-sensitive obesity. Am. J. Physiol. Endocrinol. Metab. 299, E506–E515. 10.1152/ajpendo.00586.2009, PMID: 20570822

[ref19] Koenen T. B. Stienstra R. van Tits L. J. de Graaf J. Stalenhoef A. F. H. Joosten L. A. B.. (2011). Hyperglycemia activates caspase-1 and TXNIP-mediated IL-1β transcription in human adipose tissue. Diabetes 60, 517–524. 10.2337/db10-0266, PMID: 21270263 PMC3028351

[ref20] Li J. Liu M. Zong J. Tan P. Wang J. Wang X.. (2014). Genetic variations in IL1A and IL1RN are associated with the risk of preeclampsia in Chinese Han population. Sci. Rep. 4:5250. 10.1038/srep05250, PMID: 24918527 PMC4052713

[ref21] Liang H. Ward W. F. (2006). PGC-1α: a key regulator of energy metabolism. Adv. Physiol. Educ. 30, 145–151. 10.1152/advan.00052.2006, PMID: 17108241

[ref22] Livak K. J. Schmittgen T. D. (2001). Analysis of relative gene expression data using real-time quantitative PCR and the 2(−Delta Delta C(T)) method. Methods 25, 402–408. 10.1006/meth.2001.1262, PMID: 11846609

[ref23] Lobo S. Wiczer B. M. Bernlohr D. A. (2009). Functional analysis of long-chain acyl-CoA synthetase 1 in 3T3-L1 adipocytes. J. Biol. Chem. 284, 18347–18356. 10.1074/jbc.M109.017244, PMID: 19429676 PMC2709349

[ref24] Mandal M. Donnelly R. Elkabes S. Zhang P. Davini D. David B. T.. (2013). Maternal immune stimulation during pregnancy shapes the immunological phenotype of offspring. Brain Behav. Immun. 33, 33–45. 10.1016/j.bbi.2013.04.012, PMID: 23643646

[ref25] Mao Z. Zhang W. (2018). Role of mTOR in glucose and lipid metabolism. Int. J. Mol. Sci. 19:E2043. 10.3390/ijms19072043, PMID: 30011848 PMC6073766

[ref26] McGillicuddy F. C. Harford K. A. Reynolds C. M. Oliver E. Claessens M. Mills K. H. G.. (2011). Lack of interleukin-1 receptor I (IL-1RI) protects mice from high-fat diet–induced adipose tissue inflammation coincident with improved glucose homeostasis. Diabetes 60, 1688–1698. 10.2337/db10-1278, PMID: 21515850 PMC3114387

[ref27] McGillicuddy F. C. Reynolds C. M. Finucane O. Coleman E. Harford K. A. Grant C.. (2013). Long-term exposure to a high-fat diet results in the development of glucose intolerance and insulin resistance in interleukin-1 receptor I-deficient mice. Am. J. Physiol. Endocrinol. Metab. 305, E834–E844. 10.1152/ajpendo.00297.2013, PMID: 23921145 PMC3798700

[ref28] McGowan P. O. Matthews S. G. (2018). Prenatal stress, glucocorticoids, and developmental programming of the stress response. Endocrinology 159, 69–82. 10.1210/en.2017-00896, PMID: 29136116

[ref29] Murabayashi N. Sugiyama T. Zhang L. Kamimoto Y. Umekawa T. Ma N.. (2013). Maternal high-fat diets cause insulin resistance through inflammatory changes in fetal adipose tissue. Eur. J. Obstet. Gynecol. Reprod. Biol. 169, 39–44. 10.1016/j.ejogrb.2013.02.003, PMID: 23453296

[ref30] Nair R. R. Khanna A. Singh K. (2014). Association of interleukin 1 receptor antagonist (IL1RN) gene polymorphism with recurrent pregnancy loss risk in the North Indian population and a meta-analysis. Mol. Biol. Rep. 41, 5719–5727. 10.1007/s11033-014-3443-8, PMID: 24952603

[ref31] Osuga J. Ishibashi S. Oka T. Yagyu H. Tozawa R. Fujimoto A.. (2000). Targeted disruption of hormone-sensitive lipase results in male sterility and adipocyte hypertrophy, but not in obesity. Proc. Natl. Acad. Sci. U. S. A. 97, 787–792. 10.1073/pnas.97.2.787, PMID: 10639158 PMC15409

[ref32] Plows J. F. Vickers M. H. Ganapathy T. P. Bridge-Comer P. E. Stanley J. L. Reynolds C. M. (2019). Interleukin-1 receptor-1 deficiency impairs metabolic function in pregnant and non-pregnant female mice. Mol. Nutr. Food Res. e1900770. 10.1002/mnfr.201900770, PMID: [Epub ahead of print] 31738006

[ref33] Reynolds C. M. Segovia S. A. Vickers M. H. (2017). Experimental models of maternal obesity and neuroendocrine programming of metabolic disorders in offspring. Front. Endocrinol. 8:245. 10.3389/fendo.2017.00245, PMID: 28993758 PMC5622157

[ref34] Robitaille A. M. Christen S. Shimobayashi M. Cornu M. Fava L. L. Moes S.. (2013). Quantitative phosphoproteomics reveal mTORC1 activates de novo pyrimidine synthesis. Science 339, 1320–1323. 10.1126/science.1228771, PMID: 23429704

[ref35] Saltiel A. R. Olefsky J. M. (2017). Inflammatory mechanisms linking obesity and metabolic disease. J. Clin. Invest. 127, 1–4. 10.1172/JCI92035, PMID: 28045402 PMC5199709

[ref36] Segovia S. A. Vickers M. H. Reynolds C. M. (2017). The impact of maternal obesity on inflammatory processes and consequences for later offspring health outcomes. J. Dev. Orig. Health Dis. 8, 529–540. 10.1017/S2040174417000204, PMID: 28343461

[ref37] Semple R. K. Crowley V. C. Sewter C. P. Laudes M. Christodoulides C. Considine R. V.. (2004). Expression of the thermogenic nuclear hormone receptor coactivator PGC-1alpha is reduced in the adipose tissue of morbidly obese subjects. Int. J. Obes. Relat. Metab. Disord. 28, 176–179. 10.1038/sj.ijo.0802482, PMID: 14557831

[ref38] Siljee J. E. Wortelboer E. J. Koster M. P. H. Imholz S. Rodenburg W. Visser G. H. A.. (2013). Identification of interleukin-1 beta, but no other inflammatory proteins, as an early onset pre-eclampsia biomarker in first trimester serum by bead-based multiplexed immunoassays. Prenat. Diagn. 33, 1183–1188. 10.1002/pd.4219, PMID: 23943085

[ref39] Takagi H. Ikehara T. Kashiwagi Y. Hashimoto K. Nanchi I. Shimazaki A.. (2018). ACC2 deletion enhances IMCL reduction along with acetyl-CoA metabolism and improves insulin sensitivity in male mice. Endocrinology 159, 3007–3019. 10.1210/en.2018-00338, PMID: 29931154

[ref40] Terzidou V. Lee Y. Lindström T. Johnson M. Thornton S. Bennett P. R. (2006). Regulation of the human oxytocin receptor by nuclear factor-κB and CCAAT/enhancer-binding protein-β. J. Clin. Endocrinol. Metab. 91, 2317–2326. 10.1210/jc.2005-2649, PMID: 16569740

[ref41] Weyer C. Foley J. E. Bogardus C. Tataranni P. A. Pratley R. E. (2000). Enlarged subcutaneous abdominal adipocyte size, but not obesity itself, predicts type II diabetes independent of insulin resistance. Diabetologia 43, 1498–1506. 10.1007/s001250051560, PMID: 11151758

